# Distal Acupoints Outperform Proximal Acupoints in Treating Knee Osteoarthritis: A Randomized Controlled Trial

**DOI:** 10.1155/2021/4827123

**Published:** 2021-08-18

**Authors:** Wan-Zhen Yu, Chin-Ming Huang, Hui-Ping Ng, Yu-Chen Lee

**Affiliations:** ^1^Graduate Institute of Acupuncture Science, China Medical University, Taichung City 40402, Taiwan; ^2^School of Post-Baccalaureate Chinese Medicine, China Medical University, Taichung City 40402, Taiwan; ^3^Singapore Chung Hwa Medical Institution, Singapore; ^4^Singapore College of Traditional Chinese Medicine, Singapore; ^5^Department of Acupuncture, China Medical University Hospital, Taichung City 40402, Taiwan; ^6^Chinese Medicine Research Centre, China Medical University, Taichung City 40402, Taiwan

## Abstract

**Objectives:**

To determine the difference in efficacy between distal and proximal acupoints in treating knee osteoarthritis.

**Design:**

Ninety-two eligible participants were randomly assigned into three groups: distal acupoint treatment group (DG), proximal acupoint treatment group (PG), and sham acupuncture control group (SG). Primary and secondary outcomes were compared before and after the intervention. *Interventions*. A single acupuncture treatment was applied at *Quchi* (LI11), *Shaohai* (HT3), and *Tianjing* (TE10) in DG participants; *Yanglingquan* (GB34), *Yinlingquan* (SP9), and *Heding* (EX-LE2) in PG participants; and *Zhongwan* (CV12) and *Liangmen* (ST21) in SG participants. *Main outcome measures*. The visual analog scale (VAS) and active and passive knee range of motion (ROM) were used primarily to evaluate the treatment efficacy. The radial pulse diagnosis was used as a secondary outcome measure to determine the changes in the spectral energy of the radial pulses.

**Results:**

The three groups had significant pain reduction after acupuncture (*p* < 0.05). DG had the greatest difference in pre- and post-VAS scores. Compared with the control group, significant improvement was observed in DG active and passive ROM and in PG passive ROM (*p* < 0.05). The high-frequency spectral energy of the left *chi* pulse in PG was significantly decreased, while the low-frequency spectral energy of the left *cun* pulse in PG and the left *guan* pulse in DG were significantly increased after acupuncture.

**Conclusions:**

Distal acupoints provide better pain relief and improve ROM than proximal acupoints in treating knee osteoarthritis. Significant changes in spectral energy were observed in the left *cun*, *guan*, and *chi* pulses, indicating pain relief and blood flow improvement after acupuncture.

## 1. Introduction

Knee osteoarthritis (KOA) is a common chronic progressive joint disease in aging population. The main clinical manifestations include persistent knee pain, morning stiffness, swelling, and restricted mobility. The 2018 Taiwan National Health Insurance statistics showed that 857,979 patients were diagnosed with KOA (ICD-10-CM: M17). Most patients were middle-aged and elderly above 50 years old (89.7%) [1]. A 15-year retrospective Taiwanese study reported that OA is markedly prevalent among women and increases with age noticeably [2, 3]. Moreover, the total knee replacement incidence rate tripled among patients with KOA. Some other clinical solutions include pharmacological intervention such as the use of NSAIDs, lifestyle changes, and physical treatments [4].

Acupuncture is among the common nonpharmacological treatments of KOA in Taiwan. The 2019 American College of Rheumatology/Arthritis Foundation Guideline for the Management of Osteoarthritis of the Hand, Hip, and Knee has included acupuncture among the conditional recommendations [5]. Some systematic reviews of randomized controlled trials (RCTs) related to acupuncture in KOA have concluded that acupuncture can reduce pain and improve knee function compared with sham acupuncture [6]. To date, studies comparing the acupuncture effect between distal and proximal acupoints are lacking. Therefore, we investigated the differences in pain reduction and improvement in knee function primarily through the visual analog scale (VAS) and knee range of motion (ROM). Also, objective assessment of spectral energy (SE) in radial pulses through a pulse sphygmograph was performed as a secondary outcome measure.

**Figure 1:**
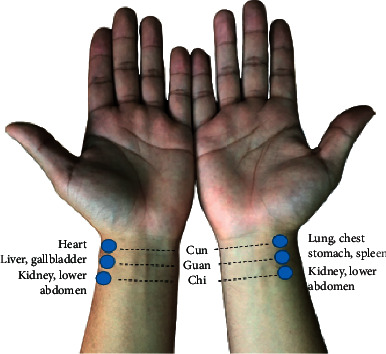
Pulse diagnosis in TCM illustrating the relationship between radial pulses and the corresponding visceral organs. Right *cun* (lung, chest); right *guan* (stomach, spleen); right *chi* (kidney, lower abdomen); left *cun* (heart); left *guan* (liver, gallbladder); and left *chi* (kidney, lower abdomen).

Radial pulse diagnosis plays an important role in traditional Chinese medicine (TCM) in distinguishing the disease position, disease pathogenesis, and the disease prognosis. Also, it guides TCM physicians in clinical decision of treatment strategy and evaluation of the treatment efficacy [7]. Traditionally, TCM physicians palpate radial pulses on both wrists of the patient using the index, middle, and ring fingers concurrently or individually [8]. Three regions corresponding to these fingers are named *cun*, *guan*, and *chi* (Chun, Guan, and Chy pulses were used in some studies), respectively [7].  Figure 1 shows the respective visceral organs corresponding to these pulses. In the modernization of pulse diagnosis, researchers have actively developed modern pulse diagnostic tools to quantify and digitalize pulse profiles since 1950 [8]. The radial pulse wave dynamics are often analyzed using time- and frequency-domain analyses. Frequency-domain analyses measure changes in SE. Wei et al. concluded that patients under metabolic stress or acute illnesses show large variations beyond 10 Hz of SE [9]. Several studies using frequency-domain analyses include observations of rhinitis, dyspepsia, atopic dermatitis, hypertension, colorectal cancer, and heat- and cold-stress patients [10–15].

There is a general perception that proximal acupoints near the diseased location are usually more effective. This study provides a novel understanding that distal acupoints outperform proximal acupoints in treating KOA using objective and subjective outcome measures.

## 2. Materials and Methods

### 2.1. Study Setting and Design

**Figure 2:**
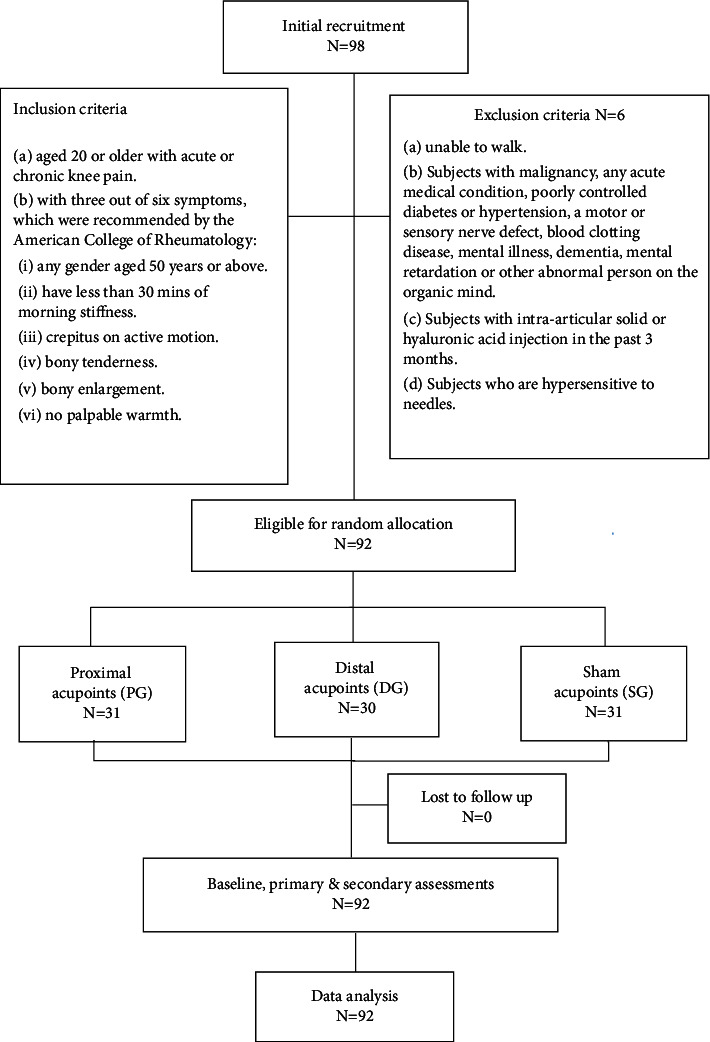
The study flow diagram.

**Figure 3:**
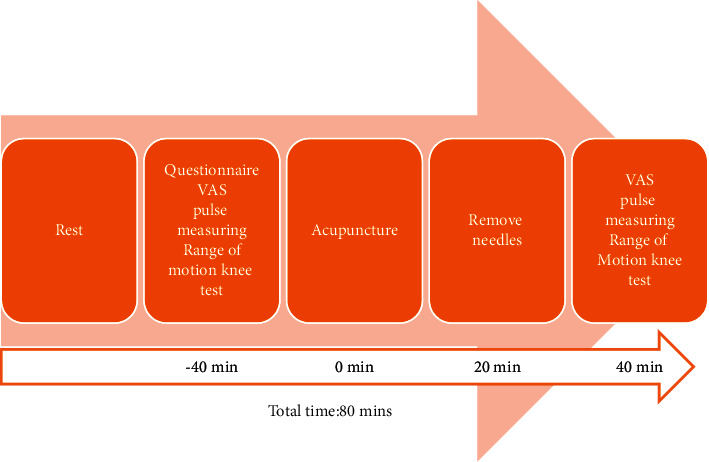
The study procedure and timeline. Each participant underwent a baseline assessment through a questionnaire. VAS, pulse assessment, and knee ROM tests were performed before and after acupuncture.

This study was conducted in the Acupuncture Department of the China Medical University Hospital (CMUH), Taichung, and Yiyuantang Chinese Medicine Clinic, Hsinchu, Taiwan, from April 2019 through March 2020. The Standard Protocol Items: Recommendations for Interventional Trials (SPIRIT) 2013 checklists were used to guide this three-arm, single-blinded, randomized study. Figures 2 and 3 illustrate the study flow and procedure.

### 2.2. Inclusion and Exclusion Criteria of Study Participants

We recruited eligible participants aged 20 or above with acute or chronic knee pain. Also, these participants must meet three out of six symptoms recommended by the ACR: (i) any gender aged 50 years or above; (ii) having less than 30 min of morning stiffness; (iii) crepitus on active motion; (iv) bony tenderness; (v) bony enlargement; and (vi) no palpable warmth. The following are the conditions that were excluded in this study: (a) participants with malignancy, any acute medical condition, poorly controlled diabetes or hypertension, a motor or sensory nerve defect, blood clotting disease, mental illness, dementia, mental retardation, or other abnormal person on the organic mind; (b) participants with intra-articular solid or hyaluronic acid injection in the past three months; (c) participants who had undergone knee surgery, knee trauma, congenital knee deformation, severe knee varus or valgus deformation, or endocrine, metabolic, infectious, inflammatory, secondary degenerative knee arthritis caused by problems with rheumatic immune diseases; (d) participants unable to walk; (e) participants who were hypersensitive to needles; and (f) participants who were unwilling to provide written informed consent.

### 2.3. Randomization

The enrolled participants were randomly assigned to three groups: distal acupoint treatment group (DG), proximal acupoint treatment group (PG), and sham acupuncture control group (SG). We used a sealed envelope system for randomization. Randomly generated treatment allocations were written on each slip and inserted into sealed opaque envelopes. The envelope was randomly picked by the patient after written informed consent was received.

### 2.4. Intervention

**Figure 4:**
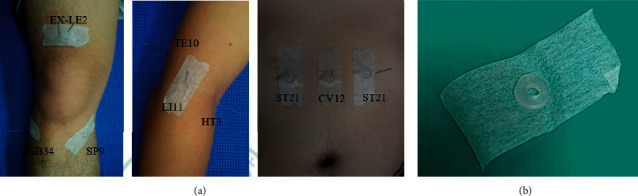
Acupoints used in this study (a) and a circular intermediate ring used (b) for all acupoints.

A single 20-minute acupuncture treatment was provided to each participant. The participants were asked to complete the Western Ontario and McMaster Universities Osteoarthritis Index (WOMAC) questionnaire in addition to the collected demographical information, including age, gender, height, weight, body mass index, and TCM meridian-related area of knee pain at baseline.  Table 1 and Figure 4 describe and illustrate the acupoints used in this study. In a meta-analysis of 18 RCTs involving KOA, GB34, SP9, and EX-LE2 were one of the common local acupoints used [17]. The distal acupoints on the upper arm, LI11, HT3, and TE10, were selected based on the holographic counterpoint theory in acupuncture. The sham acupoints at the upper abdominal region, CV12 and ST21, were selected, as there were no existing clinical studies reporting the relationship between these points and KOA treatment.

All participants were blinded in this single-blinded RCT. The acupuncture treatment was performed by a qualified TCM physician with over ten years' clinical experience. Each acupoint was located, and a circular intermediate ring adhered to the skin. Disposable, stainless steel acupuncture needles (0.25 × 40 mm; Asoon Acupuncture Needles, Taiwan) were inserted through the center of the ring for each acupoint in both DG and PG participants at the safe needling depth, and the recommended angle is described in Table 1. Moderate lift and thrust stimulations were applied while inserting and repeated after 10 min. The needles were inserted superficially without penetrating the skin through the ring for the sham acupoints. The needles were retained for 20 min.

During the acupuncture treatment, the response of the subjects was closely monitored by the qualified TCM physician. No adverse events such as acupuncture syncope, extreme pain, and hematoma were reported in all groups.

### 2.5. Study Objective and Hypothesis

This study primarily compared the immediate therapeutic effects of proximal and distal acupoints in treating KOA, including reduction of pain intensity and improvement of the knee flexibility. We hypothesized that (a) the distal and proximal acupoints may be differentially effective in relieving knee pain and improving knee flexibility and (b) the *chi* pulse will be an effective indicator for evaluating the treatment effectiveness of KOA.

### 2.6. Outcome Measures

All the assessments were performed within 40 min before and after the acupuncture (Figure 3).

#### 2.6.1. Baseline Assessments

Demographical information including age, gender, height, weight, body mass index, and affected TCM meridians distribution in addition to the WOMAC questionnaire was collected at the baseline 40 min before the acupuncture.

*(1) Western Ontario and McMaster Universities Arthritis Index*. The Western Ontario and McMaster Universities Arthritis Index (WOMAC) questionnaire is widely used to assess the symptoms and physical ability of patients with KOA or hip osteoarthritis [18]. This self-reporting questionnaire evaluates three dimensions, including pain (five questions), stiffness (two questions), and physical functions (17 questions), which can be completed within 5–10 min. An ordinal scale of zero to four was rated for each question.

*(2) Affected TCM Meridian Distribution*. Each participant was asked to identify the area of knee pain following TCM meridians and collaterals. Six options were given: lateral (Gallbladder Meridian of Foot—Shaoyang), anteromedial (Spleen Meridian of Foot—Taiyin), medial (Liver Meridian of Foot—Jueyin), posteromedial (Kidney Meridian of Foot—Shaoyin), posterior (Bladder Meridian of Foot—Taiyang), and anterolateral (Stomach Meridian of Foot—Yangming).

#### 2.6.2. Primary Outcome Measures

*(1) VAS*. The VAS is a 0-to-10 line on the left “0” (it means no pain) and on the right “10” (it means extreme pain). The patients were asked to mark their current pain levels on the line before and after the acupuncture. A 10-min rest was given before the second assessment was performed at postintervention.

**Figure 5:**
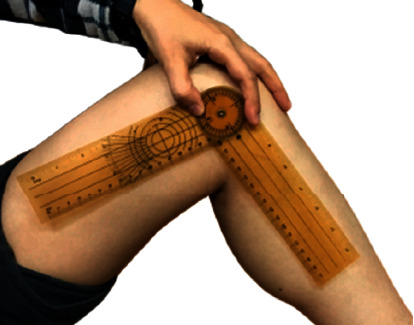
Knee ROM measured with a goniometer.

*(2) ROM*. ROM is used to determine knee joint flexibility. Active ROM and passive ROM were assessed. The active ROM is the extent of motion when the subject moves the joint voluntarily, while the passive ROM is the extent of motion when external force is applied by the investigator to move the knee. A goniometer was used to measure the angle ROM of the knee joint before and after the acupuncture (Figure 5). A 10-min rest was given before the second assessment was performed at postintervention.

#### 2.6.3. Secondary Outcome Measures

*(1) Pulse Assessment*. The acupuncture effects on the high-frequency SE (SE_13–50Hz_) in the bilateral radial pulses were assessed using a noninvasive pulse sphygmograph (Pen Pulse Analysis System Model PPAS-96; Asia Plus Biotech Co., Taiwan) before and after the intervention. This objective diagnostic tool comprised a highly precise, detachable pulse detection sensor pen with a stable Y-axial movable frame. A pulse signal analyzer containing a filter, an amplifier, and a signal-recording card was connected to collect the information for analysis. A frequency response of 0.1–50 Hz and a sampling rate of 3,000 Hz were designed in this device. The input voltage of USB_DC5V was used. The fast Fourier transform processed and digitalized the physiological signals of the radial pulses. A real-time display of the pulse spectrogram and time- and frequency-domain analyses were available as the digital output.

**Figure 6:**
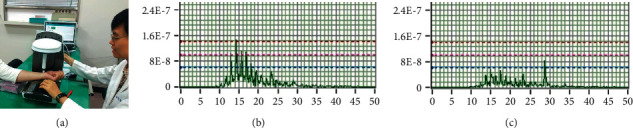
(a) Assessing each pulse on the wrist of the participants using the pulse detection sensor pen attached to the pulse sphygmograph; (b) graphical presentation of SE_13–50Hz_ of left *chi pulses* before acupuncture; and (c) graphical presentation of SE_13–50Hz_ of left *chi pulses* after acupuncture.

Each participant was seated in front of the device. *Cun*, *guan*, and *chi* pulse positions on each wrist were located and marked to ensure that same positions were subsequently repeatedly assessed at postintervention. The *guan* pulse was first identified at the prominence distal to the radial styloid process [7]. Then, the *cun* and *chi* pulses were located at the distal and proximal aspects, respectively. The assessments were performed before and after acupuncture. A 10-min rest was given before the second assessment was performed at postintervention.  Figure 6 illustrates the vertical movement of the pulse detection sensor pen at each individual pulse. The design and mechanism of PPAS-96 were described in previous studies [7, 8].

### 2.7. Sample Size

With reference to various studies related to pulse sphygmograph, a sample size of 30 participants was applied in this study. Huang et al. reported a significant difference of 0.0029 in SE_13–50Hz_ between the pre- and postintervention to compare the effects of acupuncture on radial pulse in healthy subjects and patients with dyspepsia [7, 14]. Based on the mean change and standard deviation in Huang et al.'s study, Kim et al. and Shin et al. had calculated a sample size of 25 subjects based on a 5% type 1 error, 80% power, and 5% dropout rates in their single-arm studies [19, 20].

### 2.8. Statistical Analysis

IBM SPSS Statistics V21.0 package software was used for statistical analyses. The descriptive statistics, including height, weight, WOMAC Index, VAS, and active and passive knee ROM data were analyzed. Paired *t*-tests were used to examine the spectral energy changes in the six pulses before and after acupuncture. Furthermore, one-way analysis of variance (ANOVA) was used to first investigate the preintervention results in BMI, VAS, and active and passive knee ROM between the three groups. The Scheffe method was used to identify group differences when a statistically significant difference was found. ANOVA was subsequently adopted to examine the postintervention differences in VAS and active and passive knee ROM between the three groups if no difference was found. Also, the difference in the pre-post interventions of the three groups regarding VAS, active ROM, and passive ROM was compared using ANOVA. The Scheffe method was further used to determine group differences.

### 2.9. Ethical Approval and Consent to Participate

This study was approved by the China Medical University Hospital Research Ethical Committee, Taichung, Taiwan, under protocol nos. CMUH108-REC2–033 on 10 April 2019. This study was also registered at http://www.clinicaltrials.gov under identifier: NCT03925467. We obtained written informed consent from each participant before commencing the study.

## 3. Results

Ninety-eight individuals were enrolled, but six were excluded because they were ineligible or insensitive to needles. We included 92 eligible participants who were randomly allocated to DG (30 participants), PG (31 participants), and SG (31 participants).

### 3.1. Demographic Characteristics of the Patients

The demographic characteristics, including gender, age, height, weight, and BMI, are presented in Table 2. There were 71 female and 21 male participants with an average age of 65 years. There was no significant difference in BMI, VAS, WOMAC, or active and passive knee ROM (*p* > 0.05) before the intervention when compared between the three groups using ANOVA (Table 3).

### 3.2. Affected TCM Meridian Distribution

As shown in Table 4, 76.1% of the participants suffered pain at the anteromedial aspect, while 63% suffered pain at the medial aspect of the knees, corresponding to the pathway of the spleen and liver meridians at the knee, respectively. There were often multiple meridians being affected concurrently.

### 3.3. VAS

**Figure 7:**
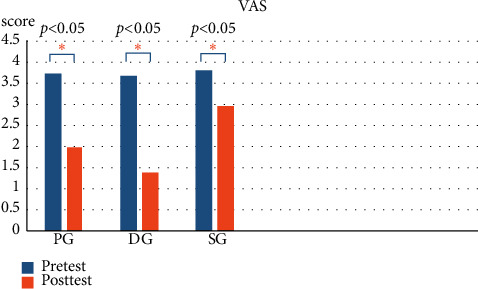
Pain scores compared before and after intervention.

Figure 7 compares the pain scores before and after the intervention. All three groups had significant differences (*p* < 0.05) in the VAS, although the pain reduction in SG is the least.  Table 5 provides the VAS differences between the three groups and post-VAS comparison. A post hoc comparison test revealed no significant difference between the PG and DG pain scores. However, significant pain reduction was found when comparing PG and SG, and DG and SG (*p* < 0.05). The greatest decrease in pain scores was observed in DG, indicating that this group of participants had the most significant improvement in pain.

Moreover, both PG-SG VAS and DG-SG VAS reached statistical significance of *p* < 0.005 and *p* < 0.001, respectively. A further step was taken to explore their Cohen's *d* effect sizes, and it was found that PG-SG VAS and DG-SG VAS differences were 0.80 and 1.05, respectively. This demonstrated that the size of difference between DG and SG VAS difference was larger than that between PG and SG VAS difference.

### 3.4. ROM

**Figure 8:**
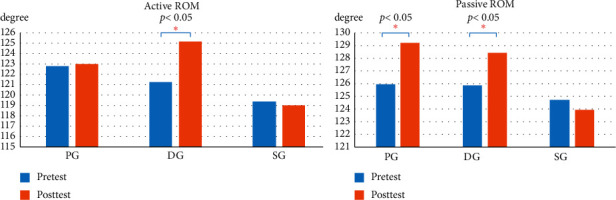
Active and passive knee ROM before and after intervention.

Figure 8 illustrates that DG showed a significant increase in knee flexibility when active and passive ROM were performed (*p* < 0.05). In contrast, PG showed the most significant increase in knee flexibility when passive ROM was performed after acupuncture.

Table 6 provides the active and passive ROM differences between the three groups, postintervention comparisons, and post hoc analysis. There were insignificant changes in the active and passive ROM differences in SG. A post hoc comparison test showed no significant difference between PG and DG passive ROM. However, marked improvement in passive knee flexibility was observed when comparing PG and SG, and DG and SG (*p* < 0.05).

### 3.5. Radial Pulse Spectral Energy

The low-frequency SE (SE_0–10Hz_) and high-frequency SE (SE_13–50Hz_) were determined in each pulse on both wrists of participants in each group. SE_13–50Hz_ significantly decreased in the left *chi* pulse, while SE_0–10Hz_ significantly increased in the left *cun* pulse after acupuncture in PG (*p* < 0.05; Table 7). In contrast, SE_0–10Hz_ significantly increased in the left *guan* pulse in DG (*p* < 0.05; Table 8). An increasing trend was observed in the SE_0–10Hz_ of right *cun* in PG and the left *cun* in DG (*p*=0.07).  Table 9 shows that no significant difference was observed in the six pulses of participants in the SG.

## 4. Discussion

### 4.1. Prevalence and Demographic Factors

Our study contained a higher proportion of female participants (77.2%) with an average age of 65 years. This agrees with the 2019 Taiwan's National Health Insurance statistics that reported that females have a higher prevalence rate, and the majority were middle-aged and elderly above 50 years old. [1] According to the recommendations from the Taiwan National Health Agency, the normal adult BMI should be maintained between 18.5 and 23.9 (kg/m^2^) [21]. We observed that all the participants were considered overweight with an average BMI above 24 kg/m^2^. A meta-analysis reported that overweight and obesity had been associated with a significantly high risk of KOA [22]. A higher score of WOMAC Osteoarthritis Index represents worse health status with noticeable joint pain, stiffness, functional limitations, and impact on quality of life [23]. An index score of ≤70% indicates mild disability (low risk), while that of >70% is noted as severe disability (high risk) [24]. The average score in each group was 28–34%, representing that all participants had mild disability.

### 4.2. Effects of Acupuncture on Pain Reduction and Knee Flexibility

The findings in pain reduction and knee flexibility suggest that our primary hypothesis is true; that is, the distal and proximal acupoints are differentially effective in relieving knee pain and improving knee flexibility.

A decrease in the VAS score is generally correlated with the effectiveness of acupuncture at proximal and distal acupoints. Marked pain reduction was observed in PG and DG patients after acupuncture in this study. Studies have revealed the immediate analgesic effect within 30 min of acupuncture treatment [25]. Some animal studies have concluded that acupuncture reduces visceral pain and induces distinct changes in neuronal activity, including the concentration level of cytokines and neurotransmitters that are related to pain or inflammation in the brain-gut axis [26]. Interestingly, the VAS score most significantly decreased in the DG, indicating the most distinct improvement in pain compared with the PG. We speculated that the concept of bioholographic acupuncture had played a part in this phenomenon. Bioholographic acupuncture uses the concept of “Embryo Containing Information of the Whole Organism (ECIWO)” developed during the 1980s. The principle involves the hypothesis that the whole organism is projected in a circumscribed part of body [27]. This principle has been used in auricular acupuncture, foot reflexology, Yamamoto's new scalp acupuncture, and others. According to the concept, the elbow corresponds to the knees. Acupuncturing the acupoints LI11, HT3, and TE10 around the elbow reduced pain in the knees. Moreover, *Quchi* (LI11) belongs to the Large Intestine Meridian of the Hand—*Yangming*, which has an abundant supply of *qi* and blood according to the meridian theory [28]. The Large Intestine Meridian of the Hand—*Yangming* is connected to the Stomach Meridian of the Foot—*Yangming*, which passes through the knees. We also observed a lesser significant pain reduction in the SG compared with the other two treatment groups. We suspected that a placebo effect could have contributed to this phenomenon.

The active knee ROM refers to the maximum extent of knee flexion-extension movement performed by the participants, while the passive knee ROM refers to that performed by an assessor. We observed that both proximal and distal acupoints improved joint flexibility. The distal acupoints significantly increased knee flexibility when active and passive ROM were performed, attributed to the result of greater pain reduction using the distal acupoints. In contrast, the proximal acupoints provided the most significant increase in knee flexibility when passive ROM was applied.

### 4.3. Effects of Acupuncture on Radial Pulses

Although we hypothesized that the *chi* pulse is an effective indicator for evaluating the treatment effectiveness of KOA, our findings showed that the high-frequency SE was significantly reduced in the left *chi* pulse, while the low-frequency SE was significantly increased in the left *cun* pulse when proximal acupoints were used. In contrast, the low-frequency SE was significantly increased in the left *guan* pulse when distal points were used. An increasing trend was observed in the low-frequency SE of the right *cun* and left *cun* when proximal acupoints and distal acupoints were applied, respectively.

We attempt to explain these observations using TCM theory and modern research. The ancient text, *Huang Di Nei Jing*, has documented that “Diagnose the pulses before acupuncture, treat the disease condition upon distinguishing the severity or ease of the *Qi*” [29]. This observation was evidenced by several modern studies that concluded that acupuncture can significantly affect radial pulses [10–15]. A hemodynamic study on healthy volunteers reported that acupuncture at ST36 (*Zusanli*) resulted in increased low-frequency SE, which corresponded to the increased blood flow velocity [19]. According to the pulse diagnosis theory, the left *cun* pulse represents the heart, which governs the blood, while the left *guan* pulse represents the liver, which stores blood. We therefore speculated that the significant increase in the low-frequency SE of the left *cun* and left *guan* pulses after acupuncture corresponded to improved blood flow in the heart and liver, respectively.

A significant reduction of high-frequency SE of the left *chi* pulse when local acupoints were used inferred that pain and inflammation were reduced after acupuncture. We speculated that the higher density and intensity of high-frequency SE generally occurred before acupuncture due to the increased vasomotion triggered by pain. A study on the effect of pain on the autonomic nervous system reported that vasomotion generally increased when sympathetic nervous activity was stimulated by pain [30]. This observation coincides with the study by Huang et al. in which elevated high-frequency SE was reported in heat-stress patients due to expanded peripheral arterioles and reduced peripheral resistance [15]. We speculated that GB34, SP9, and EX-LE2 could have attenuated sympathetic nerve activity, decreased affected muscle contraction, and decreased peripheral vasomotion. This agrees with the observation in the acupuncture treatment in the low back pain study conducted previously [7]. As the left *chi* pulse corresponds to the kidneys and lower extremities, a direct response was reflected when the local acupoints were acupunctured. This phenomenon was unobserved in the distal acupoints' treatment.

Interestingly, we found that the distal and proximal acupoints differentially affected the *chi* radial pulses on each wrist. For instance, the high-frequency SE decreased significantly in the left *chi* pulse but increased in the right *chi* pulse in PG. In contrast, a decreasing trend was observed in the high-frequency SE of both *chi* pulses in DG. Chuang et al. reported that the increase in high frequency is inferred to the increase of peripheral vascular movement. The high-frequency SE of the right *chi* pulse was significantly increased after the colectomy [13]. We speculated that acupuncture at local acupoints around the knees increased the local circulation. This observation agreed with Tsuchiya et al.'s study, which concluded that acupuncture increases the nitric oxide level in the treated regions, which may have contributed to the pain relief by acupuncture [31]. In contrast, the distal acupoints did not immediately affect the local blood flow of the knees. Therefore, only the high-frequency SE of the right *chi* pulses *in* the PG showed an upward trend.

According to TCM syndrome differentiation, symptoms of *yin* and blood deficiency increase with age [32]. The etiology of KOA is related to *qi* and blood insufficiency in the liver and kidney, which resulted in pain. The observed significant SE changes in the left *cun*, *guan*, and *chi* pulses coincided with the TCM theory that the left body is associated with blood disorders, while the right body is associated with *qi* disorders [33]. The *Huang Di Nei Jing* advised that “the kidneys dominated bones.” Therefore, this joint disorder was observed with significant changes in the *chi* pulses.

The sham acupoints CV12 and ST21 were on the abdomen. Although the acupuncture needles did not puncture the skin, the participants could still feel light stimuli. We observed a decreasing trend in the low-frequency SE of the right *guan* pulse corresponding to the stomach meridian.

The interval of assessing VAS, ROM, and pulse analysis was estimated 50–60 min. The participants were asked to rest for 10 min before the pre- and postintervention assessments. The immediate effect of acupuncture on the pain intensity, knee flexibility, and radial pulse waves were determined with minimal bias.

### 4.4. Speculated Underlying Mechanism for Distal Acupoints Outperforming Proximal Acupoints

Our study showed that the distal acupoints outperformed proximal acupoints in treating KOA. This result coincided with the conclusion in some studies investigating the effect of distal acupuncture. For instance, Irnich et al. concluded that acupuncture at distal acupoints could improve the flexibility and pain intensity in patients with chronic neck pain more than dry needling [34]. We speculated that the mechanism underlying the better results of distal points may also be due to the major role of nonsegmental antinociceptive systems in acupuncture analgesia as reported in previous studies. Various research studies have concluded that acupuncture stimulates the secretion of endorphin in the brain and it may influence the level of other neurotransmitters in the limbic system such as dopamine and serotonin. In addition, acupuncture may influence the concentration of pain-modulating neurotransmitters including substance P and met-enkephalin at the trigeminal nucleus in the brain and the spinal dorsal horn. This may be the basis for acupuncture treatment for pain including headache [35]. Further investigation on the mechanism is warranted.

### 4.5. Limitations

Several limitations of our study are as follows: First, this single-treatment study design could not observe a long-term maintenance effect of the proximal and distal acupoints. A design of multiple sessions of acupuncture can be considered in future studies to compare short- and long-term effects. Second, the VAS and ROM of the distal acupuncture had both significantly improved. In contrast, we observed a downward trend of high-frequency SE in the *chi* pulse of the DG participants. We speculated that the insignificant difference in SE was due to the low sample size. Future studies with larger sample size are warranted. Third, all participants had mild KOA, according to the WOMAC Osteoarthritis Index. The treatment outcomes may differ from those with severe KOA. Fourth, pulse wave measurement could be affected when the pressure and angle of the pulse detection sensor pen were inaccurately applied due to the sensitivity of the device. To address the issue of deviations that might be caused by the operational process, all measurements were performed by the same research staff with over a year operating experience. Fifth, the interference factors on the pulse sphygmograph, including hunger, fullness, sleep, exercise, even gender, weight, and age, have not been fully established. Further research is required to investigate the effect of these factors on the SE of pulses.

### 4.6. Implications for Future Practice and Research

This single-treatment study provides a good foundation for comparing the acupuncture effects of proximal and distal acupoints, which can be maintained during treatment. It would be interesting to add proteomic analysis to understand the mechanism of the curative effect of both treatment groups and the differences in the specific protein expression corresponding to the SE changes in the radial pulses.

## 5. Conclusion

Both distal and proximal acupoints are effective in treating KOA. However, distal acupoints provide better pain relief and improve ROM compared with proximal acupoints. Following TCM theory, significant changes in spectral energy were observed in the left *cun*, *guan*, and *chi* pulses, indicating pain relief and blood flow improvement after acupuncture.

## Figures and Tables

**Table 1:** Distal acupoints, proximal acupoints, and sham acupoints were used in this study.

Group	Acupoints	Location [16]	Application
Distal (DG)	*Quchi* (LI11)	On the lateral aspect of the elbow, at the midpoint of the line connecting LU5 with the lateral epicondyle of the humerus.	Bilateral, perpendicular insertion, 15–30 mm depth
	*Shaohai* (HT3)	On the anteromedial aspect of the elbow, just anterior to the medial epicondyle of the humerus, at the same level as the cubital crease.	Bilateral, perpendicular insertion, 15–30 mm depth
	*Tianjing* (TE10)	On the posterior aspect of the elbow, in the depression 1 B-cun proximal to the prominence of the olecranon.	Bilateral, oblique insertion (30°), 15–30 mm depth

Proximal (PG)	*Yanglingquan* (GB34)	On the fibular aspect of the leg, in the depression anterior and distal to the head of the fibula.	Bilateral, perpendicular insertion, 15–30 mm depth
	*Yinlingquan* (SP9)	On the tibial aspect of the leg, in the depression between the inferior border of the medial condyle of the tibia and the medial border of the tibia.	Bilateral, perpendicular insertion, 15–30 mm depth
	*Heding* (EX-LE2)	Above the knee, in the depression of the midpoint of the superior patellar border.	Bilateral, oblique insertion (30°), 15–30 mm depth

Sham (SG)	*Zhongwan* (CV12)	On the upper abdomen, 4 B-cun superior to the center of the umbilicus, on the anterior median line.	Unilateral, perpendicular nonpenetrating insertion.
	*Liangmen* (ST21)	On the upper abdomen, 4 B-cun superior to the center of the umbilicus, 2 B-cun lateral to the anterior median line.	Bilateral, perpendicular nonpenetrating insertion.

**Table 2:** Demographic characteristics of the study participants.

	All	PG	DG	SG
Male	21	10	6	5
Female	71	21	24	26
Age (years)	65.32 ± 10.02	64.84 ± 10.14	64.73 ± 9.57	66.35 ± 10.56
Height (m)	1.59 ± .08	1.60 ± .08	1.58 ± .07	1.58 ± .08
Weight (kg)	63.00 ± 12.66	62.55 ± 11.86	63.18 ± 13.50	63.26 ± 13.01
BMI (kg/m^2^)	24.91 ± 4.30	24.41 ± 4.69	25.09 ± 4.12	25.25 ± 4.15

Values are presented as mean ± SD. PG = proximal acupoints group; DG = distal acupoints group; SG = sham acupoints group; BMI = body mass index.

**Table 3:** Comparing the differences in BMI, WOMAC, VAS, and active and passive knee ROM in the three groups before the intervention using ANOVA.

	PG	DG	SG	*p* value
BMI (kg/m^2^)	24.41 ± 4.69	25.09 ± 4.12	25.25 ± 4.15	0.72
WOMAC (%)	27.39 ± 16.99	28.4 ± 16.97	32.58 ± 18.58	0.47
VAS	3.74 ± 1.44	3.67 ± 1.67	3.81 ± 1.82	0.95
Active ROM (°)	122.84 ± 11.89	121.27 ± 10.41	119.42 ± 13.01	0.53
Passive ROM (°)	126 ± 10.71	125.87 ± 8.65	124.71 ± 11.53	0.87

Values are presented as mean ± SD. PG = proximal acupoints group; DG = distal acupoints group; SG = sham acupoints group; BMI = body mass index.

**Table 4:** Affected TCM meridian distribution in the participants.

Group	Gallbladder (%)	Spleen (%)	Liver (%)	Kidney (%)	Bladder (%)	Stomach (%)
All	29.3	76.1	63.0	18.5	16.3	47.8
PG	19.4	77.4	64.5	29.0	19.4	54.8
DG	23.3	86.7	60.0	6.7	6.7	50.0
SG	45.2	64.5	64.5	19.4	22.6	38.7

PG = proximal acupoints group; DG = distal acupoints group; SG = sham acupoints group.

**Table 5:** VAS differences between the three groups, post-VAS comparisons, and post hoc analysis.

	PG	DG	SG	*p* value (PG vs DG vs SG)	Post hoc tests
Post-VAS	2.00 ± 1.63	1.40 ± 1.67	2.97 ± 2.06	0.001	SG > DG
VAS difference	−1.74 ± 1.12	−2.27 ± 1.55	−0.84 ± 1.13	0.001	PG > SG; DG > SG

Values are presented as mean ± SD. PG = proximal acupoints group; DG = distal acupoints group; SG = sham acupoints group.

**Table 6:** Active and passive ROM differences between the three groups, postintervention comparisons, and post hoc analysis.

	PG	DG	SG	*p* value (PG vs DG vs SG)	Post hoc tests
Postintervention active ROM	122.97 ± 23.29	125.20 ± 6.96	119.03 ± 12.56	0.31	Not applied
Active ROM difference	0.13 ± 16.38	3.93 ± 5.64	−0.39 ± 3.40	0.21	Not applied
Postintervention passive ROM	129.23 ± 11.67	128.43 ± 7.53	123.94 ± 11.03	0.10	Not applied
Passive ROM difference	3.23 ± 5.21	2.57 ± 4.08	−0.77 ± 3.53	0.001	DG > SG; PG > SG

Values are presented as mean ± SD. PG = proximal acupoints group; DG = distal acupoints group; SG = sham acupoints group.

**Table 7:** Spectral energy parameters of the radial pulse wave in PG.

Parameter	Position	Preintervention	Postintervention	*p* value
SE_0–10Hz_	Left *cun*	2.898*E* − 10 ± 2.133*E* − 10	3.559*E* − 10 ± 2.479*E* − 10	**0.03**
	Left *guan*	2.629*E* − 10 ± 1.768*E* − 10	2.562*E* − 10 ± 1.638*E* − 10	0.82
	Left *chi*	2.046*E* − 10 ± 1.031*E* − 10	2.337*E* − 10 ± 2.349*E* − 10	0.43
	Right c*un*	3.041*E* − 10 ± 2.502*E* − 10	3.679*E* − 10 ± 2.304*E* − 10	0.07
	Right *guan*	2.691*E* − 10 ± 1.805*E* − 10	3.324*E* − 10 ± 2.576*E* − 10	0.08
	Right *chi*	1.947*E* − 10 ± 1.260*E* − 10	2.124*E* − 10 ± 1.775*E* − 10	0.52

SE_13–50Hz_	Left *cun*	9.688*E* − 14 ± 2.108*E* − 13	9.788*E* − 14 ± 1.395*E* − 13	0.97
	Left *guan*	1.101*E* − 13 ± 2.313*E* − 13	8.375*E* − 14 ± 1.322*E* − 13	0.24
	Left *chi*	1.017*E* − 13 ± 1.781*E* − 13	5.891*E* − 14 ± 9.056*E* − 14	**0.047**
	Right c*un*	1.288*E* − 13 ± 2.502*E* − 13	1.455*E* − 13 ± 3.827*E* − 13	0.63
	Right *guan*	1.967*E* − 13 ± 4.383*E* − 13	1.881*E* − 13 ± 3.536*E* − 13	0.74
	Right *chi*	8.860*E* − 14 ± 1.228*E* − 13	1.551*E* − 13 ± 4.413*E* − 13	0.40

Values are presented as mean ± SD. SE = spectral energy; *E* = exponential notation.

**Table 8:** Spectral energy parameters of the radial pulse wave in DG.

Parameter	Position	Preintervention	Postintervention	*p* value
SE_0–10Hz_	Left *cun*	3.144*E* − 10 ± 2.521*E* − 10	3.871*E* − 10 ± 2.777*E* − 10	0.07
	Left *guan*	2.449*E* − 10 ± 1.598*E* − 10	3.243*E* − 10 ± 2.697*E* − 10	**0.02**
	Left *chi*	2.738*E* − 10 ± 2.062*E* − 10	2.405*E* − 10 ± 1.775*E* − 10	0.37
	Right c*un*	2.884*E* − 10 ± 1.872*E* − 10	3.643*E* − 10 ± 3.021e-10	0.11
	Right *guan*	3.441*E* − 10 ± 3.694*E* − 10	3.547*E* − 10 ± 2.799*E* − 10	0.86
	Right *chi*	3.025*E* − 10 ± 2.416*E* − 10	2.756*E* − 10 ± 2.091*E* − 10	0.43

SE_13–50Hz_	Left *cun*	1.313*E* − 13 ± 1.894*E* − 13	1.196*E* − 13 ± 1.468*E* − 13	0.74
	Left *guan*	9.398*E* − 14 ± 1.216*E* − 13	1.279*E* − 13 ± 1.732*E* − 13	0.10
	Left *chi*	1.515*E* − 13 ± 2.734*E* − 13	1.181*E* − 13 ± 2.057*E* − 13	0.12
	Right c*un*	1.865*E* − 13 ± 2.833*E* − 13	1.500*E* − 13 ± 2.714*E* − 13	0.17
	Right *guan*	2.307*E* − 13 ± 3.367*E* − 13	2.000*E* − 13 ± 3.660*E* − 13	0.68
	Right *chi*	1.391*E* − 13 ± 1.936*E* − 13	1.009*E* − 13 ± 1.317*E* − 13	0.24

Values are presented as mean ± SD. SE = spectral energy; *E* = exponential notation.

**Table 9:** Spectral energy parameters of the radial pulse wave in SG.

Parameter	Position	Preintervention	Postintervention	*p* value
SE_0–10Hz_	Left *cun*	3.405*E* − 10 ± 2.691*E* − 10	3.219*E* − 10 ± 2.017*E* − 10	0.67
	Left *guan*	2.927*E* − 10 ± 2.351*E* − 10	3.126*E* − 10 ± 2.462*E* − 10	0.57
	Left *chi*	2.606*E* − 10 ± 2.213*E* − 10	2.647*E* − 10 ± 2.006*E* − 10	0.84
	Right c*un*	3.633*E* − 10 ± 2.096*E* − 10	3.451*E* − 10 ± 2.162*E* − 10	0.63
	Right *guan*	3.178*E* − 10 ± 2.007*E* − 10	3.187*E* − 10 ± 2.770*E* − 10	1.00
	Right *chi*	2.545*E* − 10 ± 1.651*E* − 10	2.378*E* − 10 ± 1.827*E* − 10	0.56

SE_13–50Hz_	Left *cun*	1.291*E* − 13 ± 1.663*E* − 13	1.030*E* − 13 ± 1.543*E* − 13	0.24
	Left *guan*	1.463*E* − 13 ± 1.670*E* − 13	1.188*E* − 13 ± 1.323*E* − 13	0.30
	Left *chi*	1.191*E* − 13 ± 1.454*E* − 13	1.157*E* − 13 ± 1.834*E* − 13	0.87
	Right c*un*	2.377*E* − 13 ± 4.028*E* − 13	1.720*E* − 13 ± 2.490*E* − 13	0.14
	Right *guan*	2.725*E* − 13 ± 3.287*E* − 13	2.503*E* − 13 ± 3.823*E* − 13	0.42
	Right *chi*	1.425*E* − 13 ± 1.464*E* − 13	1.209*E* − 13 ± 1.262*E* − 13	0.43

Values are presented as mean ± SD. SE = spectral energy; *E* = exponential notation.

## Data Availability

The data used to support the findings of this study are available from the corresponding author upon request.

## Additional Points

Acupuncture provides immediate pain relief and improves knee ROM in treating knee osteoarthritis. Distal acupoints provide better treatment efficacy than proximal acupoints. Radial pulse diagnoses can provide an objective assessment of the acupuncture effect using a pulse sphygmograph.
